# An approach for the analysis of axonal neuroinflammation by measuring dual biomarkers of oligodendrocytes and inflammatory cytokine in human plasma

**DOI:** 10.21203/rs.3.rs-3997676/v1

**Published:** 2024-03-13

**Authors:** Masato Mitsuhashi, Akihiro Hirata, Yuko Oguma, Hiroyuki Ishida, Keisuke Kawata

**Affiliations:** NanoSomiX Inc; Keio University; Keio University; Keio University; Indiana University

**Keywords:** Neuroinflammation, subconcussion, extracellular vesicles, oligodendrocytes, interleukin 1B, myelin oligodendrocyte glycoprotein

## Abstract

The myelin sheath surrounding axons is vulnerable to mechanical stresses after head injuries, as well as autoimmune attacks and degeneration in neurological disorders. Unfortunately, there is currently no effective method to assess these axonal conditions in individual patients.

We have developed a sandwich immunoassay detecting dual signals of myelin oligodendrocyte glycoprotein (MOG) and interleukin 1B (IL1B) in human plasma ([IL1B on MOG]). While IL1B is one of common inflammation markers, its lack of tissue specificity is addressed by identifying IL1B on extracellular vesicles from oligodendrocytes isolated using anti-MOG, suggesting inflammation around axons.

In 77 control subjects, plasma levels of [IL1B on MOG] did not increase more than 2 fold from baseline. During the sports season, 14% (151 football players) and 22% (18 rugby players) exhibited a substantial 2–17 fold increase, despite the absence of traumatic brain injuries. This elevation demonstrated a non-random pattern, with some individuals gradually rising towards the season’s end, followed by a decline. [IL1B on MOG] levels also correlated with the clinical course of a post-concussion syndrome case. These data indicate that [IL1B on MOG] blood test is a potential marker for assessing mild axonal neuroinflammation.

## Introduction

**Figure 1:**
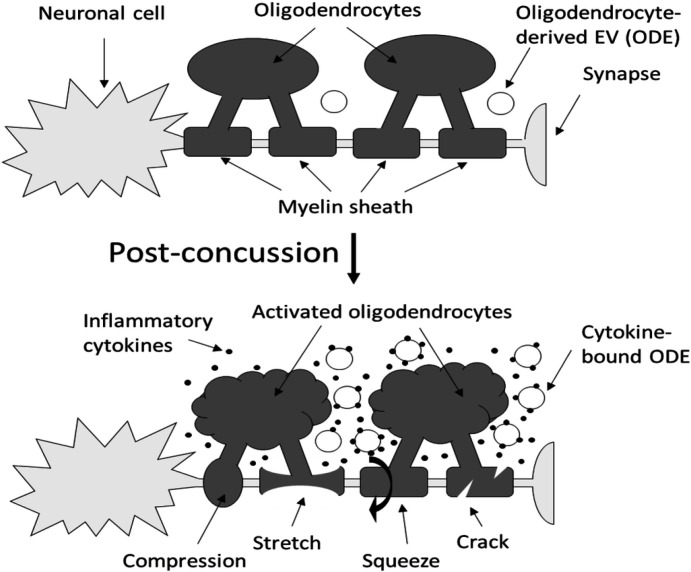
Concept of post-concussion axonal neuroinflammation assay. **Top:** The axon extends from a neuronal cell to the synaptic end and is enveloped by a myelin sheath, which is produced and regulated by oligodendrocytes (ODC). ODC release exosomes or extracellular vesicles (EV) (ODC-derived EV, ODE) into the microenvironment, subsequently entering the bloodstream. **Bottom**: After a mild head impact, the neuronal cell survives due to the flexibility of its cell body. However, the rigidity of the myelin sheath leads to damage (compression, stretch, squeeze, crack, etc.), triggering ODC activation. Activated ODCs release more ODE. In the presence of neuroinflammation, various inflammatory cytokines are released around damaged axonal lesions, with some cytokines binding to the surface of ODE. Detecting and quantifying cytokine-bound ODE in peripheral blood allows for the assessment of axonal neuroinflammation through a blood test.

Axons are long fibers conducting nerve impulses from nerve cells to synaptic ends (Fig. 1). Like electric wires, axons are insulated by the myelin sheath produced by oligodendrocytes (ODC) in the brain (Fig. 1) or Schwann cells in the periphery. The myelin sheath is vulnerable to mechanical stresses after head injuries (1–2), as well as targets for autoimmune attack in multiple sclerosis (3) and degeneration in various white matter diseases (4). Although axonal damages can be assessed by animal models or post-mortem autopsy, we do not have appropriate means available to assess and monitor such axonal conditions in individual patients. Advanced imaging analysis of computed tomography (CT) and magnetic resonance imaging (MRI) are not sensitive enough to identify such microscopic abnormalities.

Our rationale, depicted in Figure 1, highlights the contrasting properties of neuronal cell bodies and axons. Neuronal cell bodies’ pliability enables them to withstand mild mechanical forces, whereas axons, shielded by the protective myelin sheath, exhibit greater rigidity, rendering them more vulnerable to compression, stretch, squeeze, crack, etc. (1–2) (Fig. 1 bottom). Following axonal damage, ODC activation becomes instrumental in orchestrating the repair process, because ODC are brain’s sole cells capable of producing myelin. Activated ODC release a spectrum of cytokines (5), which play a vital role in activating neighboring neurons, astrocytes, and microglia, forming a complex network that facilitates repair. Activated neurons and glia cells further release cytokines (6–7), and these cytokines can recruit immune cells from the peripheral blood, triggering what is known as “neuroinflammation” (Fig. 1 bottom, small dots).

Brain cells are known to release exosomes or extracellular vesicles (EV) containing essential biomolecules derived from brain cells, and such EV are migrating into blood stream (8). This presents a unique opportunity for non-invasive evaluation, akin to a liquid biopsy. When neuroinflammation is induced around damaged axons (axonal neuroinflammation), a portion of released cytokines binds to the surface of nearby ODC-derived EV (ODE), as EV possess adhesive properties capable of absorbing various biomolecules (Fig. 1 bottom). This intriguing phenomenon suggests that by monitoring changes in cytokine profiles on ODE, we may be able to track the progression or recovery of axonal neuroinflammation in real-time through a simple blood test. While cytokines themselves are not cell or tissue-specific, their presence on ODC surface implies brain-specificity.

Validation of ODE in each clinical sample is extremely complicated, because ODE may fuse to other EV to make large complexes (9). Moreover, clinical samples may include large ODE with less surface biomarkers, small but high-density biomarkers, etc. Individual ODE characterization is important for basic science, but not applicable to clinical settings. In this study, we introduce a groundbreaking ODE-based blood test designed to assess axonal neuroinflammation in individual subjects.

## Results

### Assay development and validation.

#### Antibody screening.

We first screened various antibodies against oligodendrocytes, and found that anti-myelin oligodendrocyte glycoprotein (MOG) as the best capture agent. MOG is a transmembrane protein with a large extracellular domain at N-terminal. Our antibody recognizes the extracellular domain of MOG, so that antibody can bind to ODE under physiological condition without permeabilization or lysis procedure.

#### Capture antibody specificity (Fig. 2AB).

**Figure 2:**
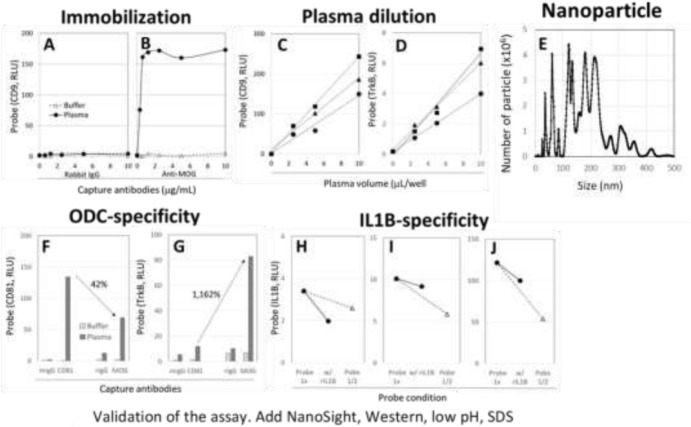
Assay validation. **A-B: Immobilization.** Various concentrations of rabbit IgG (A) and rabbit anti-human MOG were immobilized onto ELISA wells then plasma (closed circle) and plasma diluent alone (open triangle) were applied. After unbound materials were removed, biotinylated anti-human CD9 was applied to proceed to the chemiluminescent ELISA as shown in the Methods. **C-D: Plasma dilution studies.** Three different plasma samples were diluted 1/4, 1/8, 1/16 in buffer, and both diluted plasma and buffer alone were applied to the anti-MOG plate, followed by the probe reaction with anti-CD9 to quantify the amounts of captured EV (C) or anti-TrkB to determine ODC-specific signals (D). **E: Nanoparticle tracking analysis**. Plasma sample was applied to magnetic beads where anti-MOG was previously immobilized. The magnetic beads had the same surface characteristic as ELISA well, so that the same assay condition could be applied. Captured EV were eluted by a pH 2 solution, then immediately neutralized. Nanoparticle tracking analysis was performed by Particle technology Labs (Downers Grove, IL). **F-G: ODC-specificity**. Anti-CD81 (mouse IgG) and control mouse IgG (mIgG) were immobilized to ELISA wells to capture whole EVs. In the separate wells, anti-MOG (rabbit IgG) or control rabbit IgG (rIgG) were also immobilized to capture ODE and demonstrate background signals, respectively. After plasma samples (dark gray columns) and buffer alone (light gray columns) were applied, ELISA wells were exposed to anti-CD9 probes for the quantification of whole EVs (F) or anti-TrkB probes for the quantification of ODE (G). **H-J: IL1B-specificity.** Three different plasma samples were applied to anti-MOG-immobilized ELISA wells to capture ODE. Then 2 different concentrations (1x or 1/2x) of anti-IL1B probe solution was applied to show probe dose dependency. In the 3^rd^ probe solution, rIL1B was mixed with 1x probe solution.

Control human plasma or buffer alone was applied to enzyme-linked immunosorbent assay (ELISA) wells, where various concentrations of both control IgG (rabbit IgG) or anti-MOG IgG (rabbit IgG) were immobilized, followed by the probe reaction with anti-clusters of differentiation 9 (CD9). ELISA readings of relative light units (RLU) of anti-CD9 probes in the 3 controls (buffer and plasma in control IgG wells (Fig. 2A), and buffer alone in anti-MOG wells (Fig. 2B, open triangle), showed very low values compared with large increases in RLU in plasma in MOG wells (Fig. 2B, closed circle).

#### Plasma volume dilution (Fig. 2C–D).

Three different plasma samples (0, 2.5, 5, and 10 mL) were suspended in a final volume of 40 mL, and applied to MOG wells, followed by the probe reaction with anti-CD9 (Fig. 2C) or tropomyosin receptor kinase B (TrkB) (Fig. 2D), respectively. TrkB is a receptor of brain-derived neurotropic factor and is known to be expressed on the surface of ODC. Both signals showed linear dose dependent curves.

#### Confirmation of EV (Fig. 2E).

The signals of anti-CD9 on anti-MOG wells [CD9 on MOG] was reversible by exposing to pH 2 solution (data not shown). Since the amounts of captured ODE in ELISA well were very small, we immobilized anti-MOG to magnetic beads, which showed the same surface characteristics as ELISA wells. After elution of ODE, pH was neutralized then applied to nanoparticle tracking analysis to analyze size distribution. As shown in Fig. 2E, we confirmed the presence of 100–200 nm EV sized particles, as well as much larger large 300-500 nm particles, which indicate fused EV or EV aggregates.

#### ODC specificity (Fig.2FG).

TrkB is an excellent tool to demonstrate the ODC specificity on anti-MOG wells. Anti-CD81 (mouse IgG) and control mouse IgG (mIgG) were immobilized to ELISA wells to capture whole EV. In the separate wells, anti-MOG (rabbit IgG) and control rabbit IgG (rIgG) were also immobilized to capture ODE, respectively. After plasma samples and buffer alone were applied, ELISA wells were exposed to anti-CD9 probes for the quantification of captured whole EV (Fig. 2F) or anti-TrkB probes for the quantification of captured ODE (Fig. 2G). Both anti-CD81 and anti-MOG captured EV (Fig. 2F), but the amounts of captured ODE on anti-MOG wells were 42% of those of anti-CD81. However, the amounts of TrkB on anti-MOG wells were 1,162% of those of anti-CD81 (Fig. 2G), indicating over a 2,000% (20 folds) enrichment of ODE.

#### Probe specificity (Fig. 2H–J).

We then screened various cytokine biomarkers on anti-MOG wells and found that interleukin 1B (IL1B) was the best marker for this study (data not shown). In our probe solution, a huge excess volume of non-biotinylated control IgG was included. Thus, the detection of IL1B was anti-IL1B-specific. In order to further validate IL1B specificity, recombinant IL1B (rIL1B) was added to the probe solution to block anti-IL1B binding. As shown in Fig. 2H–J, anti-IL1B reaction was dose dependent in 3 different plasma (open triangle). Similarly, rIL1B decreased signals to half the amounts of the probes (Fig. 2H–J, closed circle). Thus, IL1B signals were IL1B-specific.

### Test of human samples.

#### Preliminary studies.

The target biomarker in this study was anti-IL1B signals on anti-MOG-immobilized ELISA wells [IL1B on MOG], a marker of neuroinflammation on ODE. We first tested intra-assay reproducibility, and found that the coefficient of variation (CV) was <20% (Data not shown). This is a huge benefit of this test, because we can save precious clinical samples for various analysis by running singlicate. Also, we found that serum from capillary blood is acceptable (data not shown). Then, we tested control and athlete samples using only 5 mL samples in singlicate.

#### Control values (Supplemental Fig. 1).

ELISA readings of RLU were converted to units/ml by using the dilution curve of our standard plasma, arbitrarily assigned as 100 units/mL. When we tested 63 control plasma samples, values were widely distributed over 2 logs (Supplemental Fig. 1), similar to our previous studies on neuron-derived EV (10). Thus, as shown in the next section, we focused on the longitudinal studies.

#### Controls (Fig. 3A–D).

**Figure 3:**
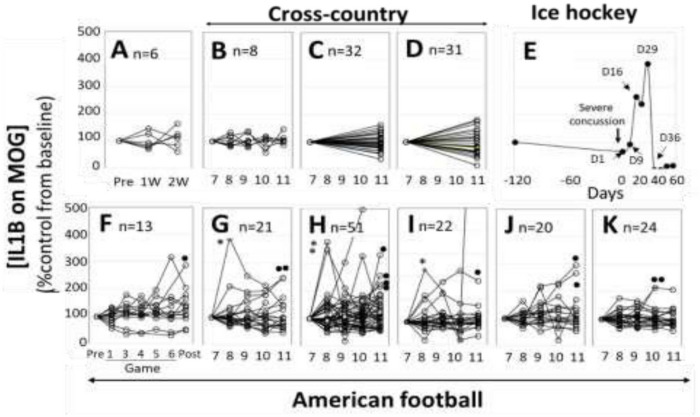
Results of human plasma samples-I. **Y axis:** %control of [IL1B on MOG] from the baseline in each subject. **X-axis**: timing of blood collection (W: week (A), 7–11: July to November (B-D, F-K) Pre and Post: July and November (F). **A: Healthy control**. Venous blood plasma samples were collected from 6 healthy adults volunteers once per week for 3 times. **B-D: Non-contact sports athletes**. Venous blood plasma samples (B) and capillary serum samples (C-D) were collected from cross-country athletes in 3 different high schools from July to November. N=8, 32, and 31, respectively. E**: Previously reported a single professional ice hockey player with severe concussion.** Venous blood plasma samples were collected from 1–54 days after head impact. Allows indicate blood collection after head impact. See reference 9. **F-K: High school American football athletes.** Venous blood plasma samples (F-G) and capillary serum samples (H-K) were collected from American football athletes in 6 different high schools from July to November. In F, blood samples were collected at each game, and in G-K, blood samples were collected regularly in each month. N=13, 21, 51, 22, 20, and 24, respectively. ·: Cases of gradual increase of [IL1B on MOG] toward the end of season. *: Cases of early increase followed by gradual decrease of [IL1B on MOG]. No concussion was reported in these athletes.

Control plasma samples (venous blood) of 6 adults were collected every week for 3 times. As shown in Fig. 3A, difference in [IL1B on MOG] from the first blood collection (%Control) was less than ± 50%. The second control was cross-country (non-contact sport) athletes from 3 different high schools (Fig. 3B–D) (n=8, 32, and 31, respectively), where no head insult was reported. Plasma samples (venous blood) were collected once every month from July to November (Fig. 3B), as well as 2 time points (July and November) of serum collection in the other 2 schools (Fig. 3C–D) from capillary blood. As shown in Fig. 3A–D, %Control of [IL1B on MOG] was all less than 200%.

#### A case study of post-concussion syndrome (PCS) (Fig. 3E).

This was a case of severe concussion of professional ice hockey player (adult male), reported previously (11). [IL1B on MOG] did not increase over the first 9 days after concussion, but substantially increased, then moved to undetectable levels after 36 days. He sustained a mild, glancing blow to the right temporal area of the head during a slow pace practice drill. He had no loss of consciousness and mild symptoms initially. Over the next few days, he began to develop a significant headache, balance issues during activities of daily living, and an aversion to loud, sudden noises. His concussion symptoms (headache and sensitivity to noise) persisted for more than a month and resolved at day 36 after concussion. MRI at day 36 showed no abnormality. As shown in Fig. 3E, [IL1B on MOG] changes were well correlated with clinical condition.

#### High school football (Fig. 3F–K) (all male).

In the 1^st^ high school football team, (Fig. 3F), plasma samples were collected by venipuncture, at each game as well as before (July) and after the season (November). In the subsequent studies, serum was collected from 5 different high schools by capillary blood once every month from July to November (Fig. 3G–K). Not all athletes showed any signs of concussion or traumatic brain injuries (TBI). As shown in Fig. 3F–K, [IL1B on MOG] stayed <200% in a majority of athletes, however, 14% showed >200% increase. All schools had at least one >200% subjects, although the incidence was varied from 8.3% (Fig. 3K) to 23.8% (Fig. 3G). More interestingly, 4 subjects (Fig. 3*) showed the increase in the 1^st^ month and gradually returned to the baseline, whereas 12 subjects showed gradual increase toward the end of the season (Fig. 3·).

#### College rugby (Fig. 4AB) (all male).

**Figure 4:**
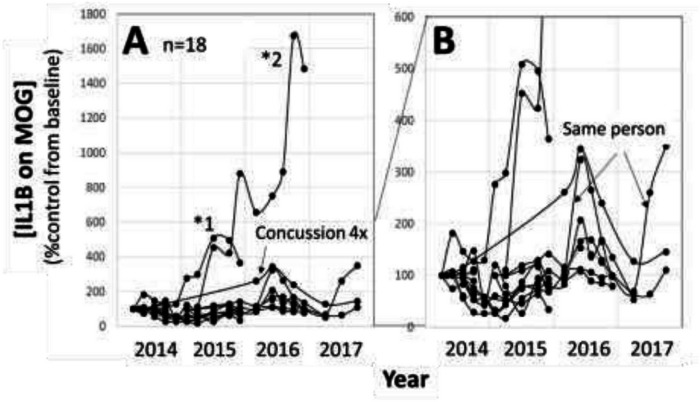
Results of human plasma samples-II. **Y axis:** %control of [IL1B on MOG] from the baseline in each subject. **X-axis**: timing of blood collection, 3–4 times/year in 18 college rugby players. **A:** Y axis from 0–1800%. *1 and *2 were players showing >400% increase in [IL1B on MOG]. Allow was the player with concussion 4 times. All other players did not have concussion episodes. **B:**Y axis scale was changed to 0–600%. Allow was a single player showing the similar pattern in both 2016 and 2017 seasons.

Plasma samples were collected by venipuncture, 3–4 times/year from 2014 to 2017 (n=18). These were the same subjects as described in our previous report (11). Since one subject showed a >16 fold (1600%) increase from the baseline (Fig. 4A, *2), the Y axis was changed from 0 to 600% in Fig. 4B. In Fig. 4A, subject #1 (*1) did not increase in 2014, but gradually increased in 2015 season. Subject #2 (*2) was striking, and gradually increased during 2015 season, then showed a much higher increase in the 2016 season. Since this person graduated in 2017, we could not follow up on his current health condition. In Fig. 4B, one person showed periodical increase in both 2016 and 2017. These 3 subjects did not show any concussion or TBI. One person showed concussion 4 times during this study period, and this person also showed >200% increase (Fig. 4A, B).

## Discussion

Inflammatory cytokines are common inflammation biomarkers, but these are not tissue-specific. In this study, we introduced a new concept of axonal lesion-specific cytokine analysis, by measuring IL1B on the surface of ODE (Fig. 1 bottom). This was possible by finding a unique feature of EV: EV surface possesses adhesive properties capable of absorbing locally released biomolecules, subsequently releasing EV into the blood stream (11–12). Although we do not know the precious mechanism of such adhesive properties, we successfully used this phenomenon and moved forward. Using previously reported sandwich immunoassay format (10–12), IL1B on ODE assay was successfully constructed (Fig. 2). While this study used a combination of 2 antibodies against MOG and IL1B in control and sports athletes, this assay is immediately applicable to other neurological disorders, where axons, myelin sheath, oligodendrocytes, and white matter are impaired by autoimmune attack such as multiple sclerosis or degenerative changes in white matter diseases, Alzheimer’s disease, Parkinson’s disease, etc. Brain cells not only secrete cytokines, but also various vital biomolecules, such as neurotransmitters, neurotropic factors, hormones, enzymes, adhesion molecules, extracellular matrix, etc. These biomolecules may be assessed by switching anti-IL1B to antibodies against these molecules. Moreover, the concept can be extended to various other inflammatory conditions, by substituting anti-MOG with antibodies against neurons, astrocytes, or non-brain cells including cancers, and targeting cytokines specific to various stages of inflammation. If the assay is complicated, labor-intensive, and requires specialized instruments, these ideas are like chasing rainbows. However, the assay platform introduced in this study is a simple ELISA with a requirement of very small volume of either venous or capillary blood. Thus, this study unveils a significant opportunity that transcends the field of neurology and inflammation.

As stated at the end of Introduction, validation of ODE in each clinical sample is extremely complicated, because ODE may fuse to other EV to make large complexes (9). In fact, we found 300–500 nm sized fused or aggregated EV in our ODE preparation (Fig. 2E). Moreover, clinical samples may include heterogeneous populations, such as large ODE with less surface biomarkers, small but high-density biomarkers, etc. Thus, we called [IL1B on MOG] without saying IL1B on ODE, and moved forward to clinical sample analysis. Since MOG is a transmembrane protein specifically expressed in the plasma membranes of ODC and Schwann cells, not freely exits in plasma. The anti-MOG antibody used in this study is specific to the extracellular domain of MOG. Thus, dual signals of both anti-MOG and anti-IL1B is likely derived from ODE as well as ODE-fused EV complex. Full ODE characterization will be completed in future.

Next question was the validation of [IL1B on MOG] as a marker of axonal neuroinflammation. As illustrated in Fig. 3–4, the pattern of [IL1B on MOG] was not random, and some showed gradual increase and return to the baseline. While the specificity of both IL1B and MOG was shown in Fig. 2, it is indeed challenging to definitively validate axonal neuroinflammation, because axonal neuroinflammation is only diagnosed at post-mortem autopsy, or wait for more than a decade to potentially witness the progression to chronic traumatic encephalopathy (CTE), or white matter dementia. Clinical samples of TBI, stroke, and various neurodegenerative disorders are the model of axonal neuroinflammation, but these are complex disorders, and various other conditions are also included. In contrast, sports athletes are young and healthy, and have few background health issues. Regular blood collection from these athletes is an ideal research model to analyze accumulated head damages. Biostatisticians may argue the statistical difference between controls and athletes, however, the main issue of this study was to demonstrate that [IL1B on MOG] really increased, not the difference between non-contact and contact sports.

Using motion sensor attached to the helmet or mouthpiece, mechanical impact can be assessed quantitatively. In fact, such motion sensors were used in our football studies (data will be available elsewhere). However, because of the difference of individual susceptibility, same degree of mechanical forces does not always induce the identical brain damages. Symptoms and physical examinations, such as dizziness, irritation to noise or lights, changes in smell or taste, numbness, etc. are based on the corresponding neuronal pathways, and show a wide individual-to-individual variation. Neurons not associated with these sensory pathways are not easily detectable using conventional physical examinations, because the brain’s inherent adaptability allows it to compensate for localized damage by finding alternate route. While this adaptability is advantageous, it also means that these concealed lesions can go unnoticed, potentially leading to future complications.

The elevation of [IL1B on MOG] was seen in some athletes who did not show concussion or TBI. This may indicate that the test may be sensitive enough to identify subconcussion or subconcussive condition (13). The term “subconcussion” or “subconcussive condition” emerged in the title of scientific papers in 2009 (14), with subsequent appearance in a few papers in each year thereafter. While these publications alarmed the public for the risk of a subconcussive condition, they did not provide practical methods for diagnosis, prediction, or assessment. Due to the lack of revealing symptoms, clinical study of subconcussion was challenging. As reported by Education News (15), the number of high school sports participants was 4.5 million male and 3.5 million female in the U.S. Furthermore, data from the National Collegiate Athletic Association (NCAA) revealed that the number of student-athletes surpass 520,000 (16). It is important to note that these statistics were U.S. alone without counting professional athletes and children who have not yet reached high school age. While the occurrence of concussion is relatively limited, potential prevalence of subconcussion or subconcussive condition is expected to be substantially higher. Subconcussion is not only the issue of athletes alone, but also a wide range of stakeholders, including families, coaches, sports doctors, teams, schools, local health departments, and policy makers, among others. Moreover, subconcussion represents a critical component within the spectrum of TBI, including falls, accidents, gunshots, blast exposures, military exercises, and more. Consequently, there is a substantial and growing demand for the diagnosis of subconcussion across diverse clinical settings.

While various assessment tools are available for the acute phase of concussion and TBI, limited resource is available for chronic consequences, where neuroinflammation develops gradually after the impact (17). In fact, in the case of typical PCS (Fig. 3E), [IL1B on MOG] did not increase immediately after concussion, and gradually increased after 9 days and decreased after 36 days, which corresponded to the clinical course in this athlete. This observation carries practical advantages, because by conducting a blood test at the time of concussion, we can establish a baseline measurement for each subject. Subsequent follow-up tests within 1–4 weeks can then provide valuable insights. If the [IL1B on MOG] levels remain unchanged during this early post-concussion period (2–4 weeks), it may suggest that neuroinflammation has not been induced, potentially providing reassurance for the athletes to return to play. Conversely, if [IL1B on MOG] levels increase within this timeframe, it may indicate the need for intervention or closer monitoring. Thus, there is compelling potential for incorporating this test into concussion guidelines and PCS management protocols in the future.

## Methods

### Reagents.

All reagents were the same as indicated in our previous publication (10), except monoclonal antibody against human MOG (Thermo Fisher Scientific, Waltham, MA) (rabbit), TrkB (R&D Systems, Minneapolis, MN) (mouse), biotinylated IL1B and rIL1B (BioLegend, San Diego, CA). Biotinylation was carried out by EZ link Sulfo-NHS-LC-Biotin (Thermo Fisher) followed by the spin column procedure to remove free biotin.

### Assay protocol.

The assay principle was a sandwich chemiluminescent ELISA with a combination of capture and detection antibodies, and detail protocol was described in our previous report (10). In this study, capture antibody was switched from anti-CD171 (neuron-specific) to anti-MOG (ODE-specific), with probes against CD9 (EV marker), TrkB (oligodendrocyte marker), and I1B (inflammatory cytokines). The RLU were determined by a luminometer (Active GLO, ANSH Labs, Webster, TX). Since the assay is 96-well format, and many samples were analyzed simultaneously, no room is available for manipulation of test results. Nanoparticle tracking analysis was carried out by Particle technology Labs, Downers Grove, IL.

### Plasma samples.

Control adult EDTA-plasma samples were purchased from 3 different commercial sources (Innovative Research, Novi, MI, BioIVT, Westbury, NY, and Equitech Enterprise, Kerrville, TX). Plasma samples from high school cross-country and American football players (Indiana University, Bloomington, IN), ice hockey (University of Louisiana at Lafayette, Lafayette, LA), and college rugby (Keio University, Kanagawa, Japan) were the same as our previous studies (10–11) with additional samples of cross-country and football from different high schools. Detail of these samples were described in the Results of those studies (10–11). For capillary blood collection, we used Tasso device (Seattle, WA) in Fig. 3B, C, D, G, H, I, J, and K). As stated in our previous report, sample collection was authorized by each institution’s Institutional Review Board or equivalent. All samples were frozen and stored in a −80°C freezer until analysis.

## Abbreviations

ODC: oligodendrocyte
CT: computed tomography
MRI: magnetic resonance imaging
EV: extracellular vesicles
ODE: oligodendrocyte-derived extracellular vesicles
MOG: myelin oligodendrocyte glycoprotein
ELISA: enzyme-linked immunosorbent assay
CD9: clusters of differentiation 9
RLU: relative light units
TrkB: tropomyosin receptor kinase B
mlgG: mouse IgG
rIgG: rabbit IgG
IL1B: interleukin 1B
rIL1B: recombinant IL1B
[IL1B on MOG]: anti-IL1B on anti-MOG-immobilized ELISA wells
CV: coefficient of variation
PCS: post-concussion syndrome
TBI: traumatic brain injuries
CTE: chronic traumatic encephalopathy
NCAA: National Collegiate Athletic Association
